# Evaluating the impact of surgery sequence on infection rates in hip or knee arthroplasty: does sequence matter?

**DOI:** 10.1007/s00264-024-06317-y

**Published:** 2024-09-17

**Authors:** Pakpoom Ruangsomboon, Onlak Ruangsomboon, Sebastian Tomescu, Cristal Rahman, Daniel Pincus, Bheeshma Ravi

**Affiliations:** 1 Sunnybrook Health Sciences Centre, University of Toronto, Toronto, ON Canada; 2 Faculty of Medicine, Siriraj Hospital, Mahidol University, Bangkok, Thailand; 3 Upstream Lab, Li Ka Shing Knowledge Institute, MAP Centre for Urban Health Solutions, Unity Health Toronto, Toronto, ON Canada; 4 ICES, Toronto, Canada

**Keywords:** Sequence, Infection, Arthroplasty, Hip replacement, Knee replacement, After

## Abstract

**Purpose:**

The potential influence of surgical sequence of elective hip-and-knee reconstructive surgery in relation to an infection-related procedure on postoperative infection rates is not clearly understood. Therefore, we aimed to examine the impact of surgical sequence on the incidence of postoperative infections within one-year and the longest available follow-up period in patients undergoing hip-and-knee reconstructive surgery.

**Methods:**

A case-control study with propensity matching was utilized to examine elective surgeries conducted at Sunnybrook Holland Orthopaedic & Arthritic centre, Toronto, Canada between 2015 and 2018. We determined and categorized them based on their operating room (OR) sequence in relation to an infected case; the cases were those performed right after (post-infection cohort), and the controls were those performed before an infection-related procedure in the same OR (pre-infection cohort). We employed survival analysis to compare the infection incidence within one year and at the longest available follow-up among the propensity-matched cohort.

**Results:**

A total of 13,651 cases were identified during the four year period. We successfully matched 153 cases (21 post-infection and 132 pre-infection) using propensity scores. Demographic and clinical characteristics were balanced through matching. Kaplan-Meier survival analysis showed no significant difference in infection-free survival within one year and at a median follow-up of 2.2 years [interquartile range 0.9-5.0] between surgeries conducted before and after infected cases (both log-rank p-values = 0.4). The hazard ratios for infection within one year and the longest follow-up period were both 0.37 [95%Confidence Interval 0.03–4.09, *p* = 0.418], as no more events occurred after one year.

**Conclusion:**

The sequence of surgical procedures, whether or not an elective arthroplasty or lower limb reconstructive procedure occurs before or after an infection-related case in the same OR, does not significantly affect postoperative infection rates. This finding supports the efficacy of the current infection control measures and suggests a reconsideration of surgical scheduling standards.

## Take home message

- The sequence of surgical procedures, whether performed after an infected case or not, might not affect the rate of postoperative infections within the first year and until the median follow-up time of 2.2 years after surgery.

## Introduction

Postoperative infections, both deep and superficial, significantly compromise patient outcomes and place a substantial clinical and economic burden on healthcare systems worldwide [1]. The aetiology of such infections is multifaceted, with contributing factors ranging from patient, surgical and environmental factors [2, 3]. Many patient-related factors contribute to developing infection after surgery. Modifiable patient factors can be directed at to minimize peri-prosthetic joint infection (PJI) risks, such as diabetes control, optimizing patient nutrition and appropriate surgical site self-preparation [4]. Surgical factors, especially the surgical team’s aseptic protocol using sterile technique, are also mandatory.

The surgical process should be carefully choreographed to prevent intra-operative contamination, which can range from precise sterilization of instruments to scrupulous application of hand scrubs. The operating room (OR) environment itself, influenced by factors such as room cleaning protocol, air flow, and personnel traffic, is also a known determinant of infection risk. Innovations including ultraviolet light technology and laminar airflow systems have been implemented to reduce bacterial counts and optimize sterile conditions [3, 5].

Despite these advances, there remains a scarcity of robust scientific evidence on one aspect of environmental factors – the sequence of surgeries – and its effect on postoperative infection rates. This notion stems from concerns that arthroplasty or any elective surgery performed after an infected case might elevate the risk of postoperative complications. In 2017, Chen et al. reported an increased risk of infection for joint arthroplasties following infected cases on the same day in the same OR [6]. In contrast, Abolghasemian et al. did not find an increase in infection risk when subsequent arthroplasty surgeries were performed in the OR after infected cases [7]. These conflicting results highlight the need for further studies on the influence of surgical sequence on post-operative infection.

We hypothesized that the sequence of surgical operations may not increase the rate of postoperative infection if the team strictly adhere to proper patient care protocols, sterile surgical procedures, and appropriate environmental sanitation practices. Our primary objective was to compare the rate of superficial and deep infections occurring within one year after the index surgery between elective hip-knee surgical cases performed before and after an infected case in the same OR. The secondary objective was to investigate the impact of the surgical sequence on the probability of infection over the longest available follow-up period.

## Methods

### Study design and setting

This study was a single-, case-control study conducted at Sunnybrook Holland Orthopaedic & Arthritic centre, the largest joint arthroplasty centre in Canada. The majority of the procedures performed at this facility were elective surgeries of hip or knee arthroplasty. This study was approved by the institutional review board (REB- CR6108), and patient consent was waived due to the retrospective nature of the study. Given the infrequent occurrence of surgeries after infected cases, a cases-control approach was utilized within the cohort of every patient in the hospital database between 5th January 2015 and 21st December 2018.

### Participants

This study involved a detailed examination of OR scheduling logs during the four-year period from 2015 to 2018 to identify cases of scheduled elective joint arthroplasty, osteotomy, infected cases, and arthroscopic surgery. The initial step involved identifying surgical cases with interventions indicative of the presence of an infection, which included procedures such as debridement of infected surgical wound, incisional and drainage, arthrotomy with debridement, debridement with implant retention, first-stage debridement, and amputation due to unmanageable infection.

Subsequently, we identified and included into the study cohort patients undergoing elective arthroplasty or surgery of lower extremities classified as clean-wound procedures, namely corrective osteotomy, arthroscopic surgery, and patellofemoral reconstructive surgery, which were performed before and after the index infection-related procedure. Cases associated with history of infection including second-stage revision following initial stage debridement of PJI were excluded. Included patients were then divided into two distinct cohorts, the cases and the controls, based on their sequence in relation to the infected cases. Cases were any elective clean-wound surgeries done after an infected case in the same OR on the same day, to which we referred as the “post-infection sequence cohort”. Conversely, the controls were clean-wound procedures performed before the first case of infection in the same OR on the same day, a more typical scenario that we categorized as the “pre-infection sequence cohort”.

### Data collection and outcomes

The OR scheduling logs and follow-up results were reviewed by three surgeons [PR, ST, BR]. Each case was independently assessed, and cross-verified among the team members to ensure the reliability and validity of the data. Patients’ baseline characteristics, particularly those influencing postoperative infections [3, 4] including cancer, diabetes mellitus, genitourinary disease, hepatobiliary disease, neurological diseases including Alzheimer’s disease and Parkinson’s disease, obesity, psychological disease including depression, active smoking, and peripheral vascular disease, were assessed via the hospital database.

The primary outcome was any superficial or deep infection occurring within one year after the index surgery. Superficial wound infection, as defined by the Surgical Infection Study Group (SISG), encompasses the presence of pain, edema, erythema, warmth, and impaired function around the surgical site [8]. Deep infection was defined as an infection that persists after surgery and necessitates any debridement or surgical intervention into the previous surgical area, in addition to antibiotic treatment. This study primarily focused on short-term one-year infection rates as the primary outcome because existing literature demonstrates that most infections related to microorganism from intraoperative contamination occur within this timeframe [9]. The secondary outcome was an infection occurring over the longest available follow-up time. Participants’ medical records were reviewed and documented in March-April 2024 by one of the three study surgeons for notes indicative of the presence of superficial or deep infection post-surgery. All instances of infection were then documented to assess the one-year postoperative infection rates. To ensure comprehensive follow-up, all participants were monitored for the most recent infections recorded in both our hospital database and the interconnected community hospital database. This approach includes verifying any infections treated elsewhere or managed with antibiotics, as reported in patients’ histories or noted from patients’ presentations at any hospital. Additionally, all email communications from patients were included in the virtual care notes within the hospital database system to ensure no details were missed.

### Statistical analysis

Categorical data are described using frequency and percentage and compared using the Chi-squared test. Continuous variables are summarized and presented as mean with standard deviation or median with interquartile range (IQR) as appropriate. They were compared using the student t-test when normally distributed. We employed propensity score matching to mitigate confounding variables and create two balanced cohorts between the cases and the controls. Utilizing R version 4.3.3, a full propensity score matching was conducted via MatchIt package [10]. We calculated a propensity score for each participant by considering significant baseline characteristics based on an absolute standardized difference of 0.2 [11]. The characteristics under consideration were age, gender, and pre-existing conditions that can increase the risk of post-operative infection, including cancer, diabetes mellitus, genitourinary disease, hepatobiliary disease, neurological disease, body mass index (BMI) over 30 kg/m^2^, psychiatric disease, smoking, and vascular disease [4]. The technique included a full matching method on propensity scores with a caliper of 0.2, based on the logit of the propensity score, to balance the aforementioned characteristics between the two cohorts. We evaluated the balance improvement by determining standardized differences for each covariate pre- versus post-matching, ensuring that any significant disparities were rectified before proceeding to the main analysis. Following this, the cohorts underwent a survivorship analysis to determine the infection rates within one year of the procedure and at the most recent follow-up. The time-to-event data are visually represented as the Kaplan-Meier survival curves through the utilization of the Survival [12] and Survminer packages [13] in R version 4.3.3. Results are reported as the hazard ratio (HR) with 95%Confidence Interval (95%CI) and the p-value from the log-rank test. A p-value of < 0.05 was considered statistically significant. There was no missing data in the present study.

## Results

### Demographic and clinical characteristics pre- and post-matching

From 2015 to 2018, our OR logs identified 13,651 elective and clean orthopedic surgeries. The annual case numbers were 3,372 in 2015, 3,387 in 2016, 3,396 in 2017, and 3,496 in 2018. Of these, only 24 met our stringent criteria as the post-infection sequence cohort, reflecting the relatively rare occurrence of elective surgeries following infected cases, while 151 cases were identified and determined as the pre-infection sequence cohort.

Before propensity score matching, age and sex distribution did not significantly differ between the pre- and post-infection sequence groups. However, certain comorbidities, notably cancer, genitourinary, and vascular diseases, were unequally distributed across the cohorts (Table 1).

**Table 1:** Demographic and clinical characteristics pre-propensity matching

Clinical variables	Pre-infection sequence (*N* = 151)	Post-infection sequence (*N* = 24)	*P*-value	Absolute standardized difference
Age [mean (SD)]	64.6 (15.0)	67.6 (8.3)	0.155	NA
Male (n, %)	61 (40.4%)	11 (45.8%)	0.780	0.11
Cancer (n, %)	4 (2.6%)	0	0.943	0.233
Diabetes Mellitus (n, %)	16 (10.6%)	3 (12.5%)	1.000	0.059
Genitourinary (n, %)	10 (6.6%)	0	0.409	0.376
Hepatobiliary (n, %)	1 (0.7%)	0	1.000	0.115
Neurological (n, %)	17 (11.3%)	2 (8.3%)	0.940	0.099
Obesity > 30BMI (n, %)	35 (23.1%)	5 (20.8%)	1.00	0.056
Psychiatric (n, %)	0	0	NA	NA
Smoking (n, %)	8 (5.3%)	1 (4.2%)	1.000	0.053
Vascular (n, %)	1 (0.7%)	3 (12.5%)	0.004	0.491

*Abbreviation* SD; standard deviation

Following propensity score matching, age means were closely aligned at 64.8 ± 14.6 years for the pre-infection sequence group and 67.1 ± 8.8 years for the post-infection sequence group (*p* = 0.332). Sex distribution post-matching showed minimal variance with 36.4% and 42.9% males in the pre- and post-infection sequence groups, respectively (*p* = 0.742). This matching process effectively neutralized comorbidity disparities as potential confounding factors, yielding all insignificant standardized differences (Table 2). The final matched cohorts comprised of 153 patients, with 21 cases and 132 controls. The 21 post-infection sequence cohort underwent operations performed after 16 infected cases in the same OR on the same day. Among these 16 cases, five were due to isolated Gram-positive organisms [*3 cases of coagulase-negative Staphylococci*,* 1 case of Methicillin-sensitive Staphylococcus aureus* (MSSA), *1 case of Methicillin-resistant Staphylococcus aureus* (MRSA)], two were due to isolated Gram-negative organisms [*1 Haemophilus parainfluenzae Biotype III* (Beta lactamase negative), *1 Proteus mirabilis*] and four were from multi-organism infections [*1 case of Coagulase-negative staphylococci and Finegoldia magna* (previously known as *Peptostreptococcus magnus*), *1 case of Enterococcus faecium and Coagulase-negative staphylococci*,* 1 case of Streptococcus mitis group and Coagulase-negative staphylococci*,* 1 case of Cutibacterium acnes* (previously known as *Propionibacterium acnes*) *and Staphylococcus epidermidis*].

**Table 2:** Demographic and clinical characteristics post-propensity matching

Clinical variables	Pre-infection sequence (*N* = 132)	Post-infection sequence (*N* = 21)	*P*-value	Absolute standardized difference
Age [mean (SD)]	64.8 (14.6)	67.1 (8.8)	0.332	NA
Male (n, %)	48 (36.4%)	9 (42.9%)	0.742	0.133
Cancer (n, %)	1 (0.8%)	0	1.000	0.123
Diabetes Mellitus (n, %)	14 (10.6%)	3 (14.3%)	0.901	0.112
Genitourinary (n, %)	0	0	NA	NA
Hepatobiliary (n, %)	0	0	NA	NA
Neurological (n, %)	15 (11.4%)	2 (9.5%)	1.000	0.060
Obesity > 30BMI (n, %)	31 (23.5%)	4 (19.0%)	0.865	0.109
Psychiatric (n, %)	0	0	NA	NA
Smoking (n, %)	0	0	NA	NA
Vascular (n, %)	0	0	NA	NA

*Abbreviation* SD; standard deviation

Table 3 summarizes the post-matching clinical characteristics, diagnosis, mean age, and sex distribution among the matched cohort. Most cases were those with osteoarthritis of the knee (50.3%), followed by osteoarthritis of the hip (31.3%). Within one year of follow-up, 97.7% (129/132) of the pre-infection sequence group and 95.2% (20/21) of the post-infection sequence group remained infection-free. Among the pre-infection sequence cohort, there were three incidences of infection, two superficial wound infection and one suture abscess. In the post-infection sequence group, only one case encountered a suture abscess. All of these cases developed an infection within six weeks after surgery and were successfully treated with an oral antibiotic. No other infection occurred in both the study groups until the latest follow-up time point, with a median follow-up time of 2.2 years (IQR 0.9-5.0 years) for both groups.

**Table 3:** Details and demographic of propensity-matched cohort

Diagnosis	*N* = 153, *n* (%)	Mean age (SD)	Male: Female	Male %
Osteoarthritis of the knee	77 (50.3)	68.1 (10.6)	21: 56	27.3%
Osteoarthritis of the hip	48 (31.3)	63.9 (14.3)	20: 28	41.7%
Aseptic loosening/ wear/ instability	14 (9.2)	67.9 (12.2)	9: 5	64.3%
Others*	14 (9.2)	50.1 (20.4)	7: 7	50.0%

*Abbreviation*: SD; standard deviation

*including extra-articular deformity, meniscus or ligament injury around the knee, patellofemoral instability

### Survivorship analysis results

**Fig. 1:**
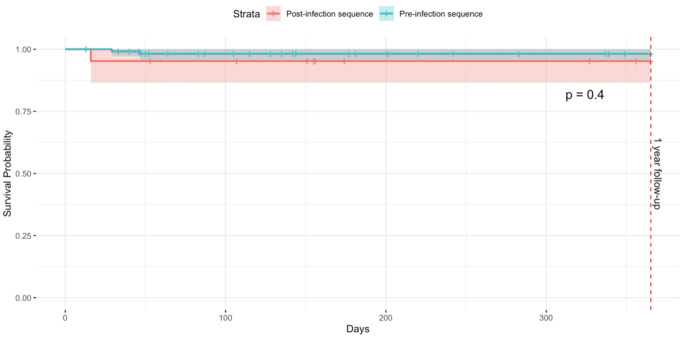
Survivorship within one year by operating room sequence

Within the first-year post-surgery, the Kaplan-Meier estimator did not demonstrate a statistically significant difference in the survival probability of being infection-free between the two cohorts (log-rank p-value = 0.4). The HR for infection occurring within one year was 0.37 [95%CI 0.03–4.09; *p* = 0.418]. This indicates that the sequence of surgery—whether it occurred before or after an infected case—did not significantly alter the probability of remaining infection-free within this period. The mean survival time for both groups remained comparably high, with overlapping confidence intervals, further suggesting that there was no discernible difference in the risk of infection (Fig. 1).

**Fig. 2:**
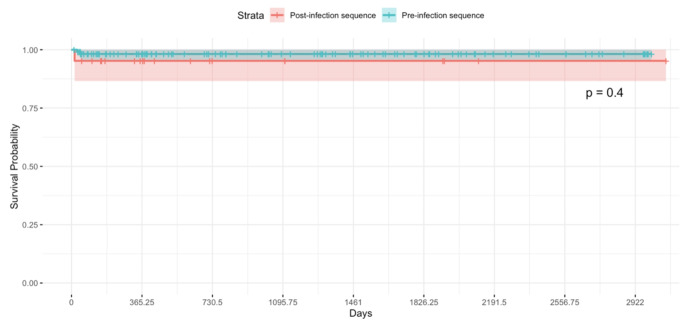
Survivorship at the latest available follow-up period by operating room sequence

For the extended follow-up period, the survival curves followed a similar trend (Fig. 2), with the same hazard ratio and p-value as those of the primary outcome, as no new events occurred after one year. Similarly, the longer-term survivorship did not significantly differ between the groups (*p* = 0.4), indicating that the sequence of surgery did not have a longer-term impact on the rate of postoperative infections.

## Discussion

Our main finding found that the sequence of surgical procedures, whether performed after an infected case or not, did not affect the rate of postoperative infections within the first year. Furthermore, patients remained free from infection until the latest follow-up, with a median follow-up time of 2.2 years after surgery. This discovery contradicts conventional wisdom and some of the previous reports that indicated a possible association between performing surgery after infected cases and the development of post-operative infection. Our findings not only call into question long-held assumptions in surgical practices about the OR sequence, but also reflect the importance of providing optimal quality of care across the patient care pathway, which could lead to effective infection control after surgery.

It is generally known that studying and understanding postoperative infection is challenging. The occurrence of post-operative infections is influenced by multiple factors. Also, ethical limitations make it challenging to carry out a robust study design such as a randomized controlled trial. Therefore, a well-designed case-control study is probably the most feasible design to investigate our research question. Our analysis spanned a broad timeframe of four years and screened a diverse cohort of 13,651 cases, enabling a comprehensive examination of the sequence effect within a unified setting. We also employed propensity score matching, a step not delineated in any previous study, which allowed for a robust comparison between groups by balancing numerous known confounding factors that may affect post-operative infection outcomes. Consequently, the present study provides a strong piece of evidence that the sequence of arthroplasty-related surgery in relation to an infected case did not affect the rate of one-year or longer-term infection.

The authors believe that, rather than the OR sequence, infection control protocols are the much more important factor influencing post-operative infection outcomes. At our institution, the routine patient care pathway spans beyond the OR. During the preoperative phase, every patient is screened and optimized for all modifiable conditions, including comorbidities management and control and promoting good nutrition through consultations with a multidisciplinary team of anesthesiologists and internal medicine specialists. In the OR, instead of relying on space suits, we adhere to meticulous hand-scrubbing routines and use disposable gowns, gloves, and sterile dress to maintain a sterile environment. Our ORs also utilize laminar airflow systems that help maintain an optimal surgical setting by reducing airborne contaminants. Additionally, our standard protocol includes copious irrigation with bulb syringe or pulsatile lavage with normal saline within the surgical area to minimize or dilute the possibility of bacterial load prior to wound closure. Routine cleaning of the OR was meticulously performed after each case by a team of three environmental service providers. The process involves one person stripping the linens, then collecting all linens and garbage bags, while the second person cleans all dirty surfaces starting with the OR table and reusable grounding pad, then moving to the anaesthesia machine, all OR tables including drill and preparation tables, OR lights, and mayo stand. Nursing and anaesthesia computer stations are also wiped down. Ceiling and walls are inspected and cleaned as necessary. The third person is responsible for mopping the entire room, including moving and cleaning under the OR table using PERdiem^®^, a hydrogen peroxide solution for mopping the floors. Oxivir Plus^®^, a 7% hydrogen peroxide solution is used for wiping all high-touch items. Clorox^®^ germicidal bleach wipes are specifically used for wiping the reusable grounding pad.

Therefore, the authors hypothesized that the culmination of these strategies affect our patients’ postoperative infection-free outcomes in the present study. In fact, the discrepancies between the findings of the study by Chen et al. [6] and our own could have been attributed to the evolution of sterilization techniques and infection control measures that have been enhanced and implemented over the recent years.

The strength of the present study lies in its methodology, ensuring that the observed outcomes are attributable to the sequence of surgeries rather than underlying patient characteristics by employing a propensity score matching accounting for a wide range of comorbidities that may affect infection outcomes. However, it is important to highlight several weaknesses. First, due to the low infection rate following arthroplasty in our institution (0.5%), the small sample size and low event rate was a potential limitation in this study, and we might have been underpowered to detect an important difference. This low rate also limits the possibility to explore the association of other potential contributors such as the effect of type of organism or bacterial profile of the infected case on the likelihood of infection in subsequent clean cases. However, we made every effort to include all cases from our centre, which has the largest patient volume in Canada, in order to create a unified setting with the highest number of participants possible. Second, although we attempted to balance known potential confounders through a propensity score matching, there could still have been unmeasured confounders unaccounted for. Third, the results of this study might not be generalizable to other centers with lower quality infection-control protocols. Consequently, the results of this study should serve as a basis informing future research or systematic reviews.

In summary, while many OR scheduling practices have abided by the notion that the sequence of surgery or the procedure performed after an infection-related case is a potential risk factor for surgical site infections, our findings introduce a new perspective, suggesting that under rigorous infection control measures, the surgical sequence may not have a clinically important impact on infection rates. This finding may advocate a reconsideration of surgical scheduling standards.

## Data Availability

Requests for data not shown in the body of this manuscript can be made to the corresponding author.
